# Recent developments in human immunodeficiency virus-1 latency research

**DOI:** 10.1099/vir.0.049296-0

**Published:** 2013-05

**Authors:** Chi Ngai Chan, Isabelle Dietrich, Margaret J. Hosie, Brian J. Willett

**Affiliations:** MRC-University of Glasgow Centre for Virus Research, Bearsden Road, Glasgow G61 1QH, UK

## Abstract

Almost 30 years after its initial discovery, infection with the human immunodeficiency virus-1 (HIV-1) remains incurable and the virus persists due to reservoirs of latently infected CD4^+^ memory T-cells and sanctuary sites within the infected individual where drug penetration is poor. Reactivating latent viruses has been a key strategy to completely eliminate the virus from the host, but many difficulties and unanswered questions remain. In this review, the latest developments in HIV-persistence and latency research are presented.

## Introduction

Before the introduction of highly active anti-retroviral therapy (HAART), a diagnosis of HIV/AIDS would have been a death sentence for most patients. However, modern anti-retroviral regimes are able to preserve the health of the patient and routinely reduce the plasma viral load to less than 50 copies of HIV-1 RNA ml^−1^ (Volberding & Deeks, 2010). Although HAART is very effective at blocking HIV-1 spread within the body, it is not a cure, as viral loads readily rebound when treatment is interrupted (Chun *et al.*, 1999; Davey *et al.*, 1999). Furthermore, ultrasensitive detection assays have shown that in most HAART-treated patients, a low-level viraemia of less than 5 copies ml^−1^ persists even after years of therapy (Chun *et al.*, 2005; Palmer *et al.*, 2008; Tobin *et al.*, 2005). This low-level persistent viraemia is a major obstacle to the complete elimination of HIV-1 from the body.

HAART cannot fully restore the health of an infected individual. Long-term-treated HIV-1 patients have reduced lifespans and increased susceptibilities to non-AIDS related conditions such as cardiovascular disease, cancer, liver and kidney dysfunctions as well as neurological decline, which may be a consequence of the toxicity of the drugs or the chronic inflammation caused by HIV-1 infection (Deeks, 2011; d’Arminio *et al.*, 2004). The financial cost of life-long treatment, especially in resource-poor settings, is prohibitive (Hecht *et al.*, 2010). Until the discovery of an effective vaccine, or other interventions that can halt the continuing spread of HIV-1, it will become increasingly difficult for high disease burden countries in the developing world to control the epidemic using only current anti-retroviral regimes (Lewin *et al.*, 2011). Thus, an effective cure of HIV/AIDS would not only alleviate the suffering of the millions of infected persons, it may be the only way to check the progress of the HIV-1 epidemic. In this article, we provide an overview of HIV-1 latency and address some of the major gaps in our understanding of the phenomenon. We examine recent advances in translational research aiming to find a sterilizing (complete eradication of the virus) or a functional (virus replication is on-going but does not lead to clinical problems) cure.

## The question over the source of the persistent viraemia

The half-life of the HIV-1 virion in the plasma is very short (Ho *et al.*, 1995; Ramratnam *et al.*, 2000) and it is generally believed that the persistent viraemia is either the result of the reactivation of latently infected resting T-cells or on-going virus replication in ‘sanctuary’ sites within the body (Lewin *et al.*, 2011; Palmer *et al.*, 2011). The existence of a latently infected population of CD4^+^ T-cells was first indicated by the discovery that the number of cells expressing HIV-1 mRNA *in vivo* was lower than the number of cells carrying proviral DNA (Schnittman *et al.*, 1989). Subsequent studies have demonstrated that a small number (approx. one million cells) of resting CD4^+^ T-cells in HAART-treated individuals harboured replication-competent latent viruses that could be reactivated by stimulation of the cells with mitogens (Chun *et al.*, 1995, 1997; Finzi *et al.*, 1997). While dormant, the virus is hidden from the host immune response and it has been shown that the decay rate of these latently infected resting CD4^+^ T-cells is very low, requiring an estimated period of 73.4 years for complete eradication using the current anti-retroviral regime (Siliciano *et al.*, 2003). Alternatively, on-going low-level virus replication may be responsible for the persistent viraemia. Persistent virus production has been found within sanctuary sites such as the central nervous system (Canestri *et al.*, 2010; Churchill *et al.*, 2006; González-Scarano & Martín-García, 2005), the gastrointestinal tract (Chun *et al.*, 2008) and the male and female genital tract (Halfon *et al.*, 2010; Launay *et al.*, 2011). Recent studies have indicated that anti-retroviral drug-penetration is site- and compound-specific, and drugs that penetrate poorly may allow virus replication at that site even when plasma viral load is below 50 copies ml^−1^ (Best *et al.*, 2012; Di Mascio *et al.*, 2009; Else *et al.*, 2011; Halfon *et al.*, 2010; Kwara *et al.*, 2008; Launay *et al.*, 2011). Since the rate of reactivation of latent viruses in resting T-cells is unknown *in vivo* (Siliciano & Siliciano, 2010), it is unclear whether it occurs frequently enough to maintain the low-level viraemia that is detected in patients. Thus, the most probable origin of the low-level viraemia may be the sanctuary sites where productive infection is expected to be occurring constantly.

In order to determine the contribution of each of these factors to the low-level viraemia in the body, phylogenetic studies were performed on the viral sequences isolated from the residual viraemia. The results were contradictory: while some studies showed a lack of evolution among the sequences found, suggesting that the progeny virions came from one stable reservoir among CD4^+^ T-cells (Bailey *et al.*, 2006; Joos *et al.*, 2008; Ruff *et al.*, 2002), others found viral sequences that were not detected among the resting T-cell population, indicating another cellular source for the residual viraemia (Bailey *et al.*, 2006; Brennan *et al.*, 2009; Sahu *et al.*, 2009). Another indication of on-going productive infection would be if treatment intensification (the addition of a fourth anti-retroviral to the standard three-drug regime) reduced the basal level of viraemia further. The majority of treatment intensification studies using the HIV-integrase inhibitor Raltegravir (RGV) showed no significant reduction of the residual plasma viraemia (Dinoso *et al.*, 2009b; Gandhi *et al.*, 2010; Hatano *et al.*, 2011; McMahon *et al.*, 2010). However, in one study RGV increased the number of ‘2-LTR (long terminal repeat) circles’ found in the PBMCs of 29 % of the treated subjects (Buzón *et al.*, 2010). Since RGV blocks the integration of linear viral DNA from a productive infection and encourages the formation of 2-LTR circles (Middleton *et al.*, 2004; Svarovskaia *et al.*, 2004), these data indicate the presence of an on-going infection. In a separate study, intensification with RGV reduced unspliced HIV-1 RNA within the ileum, but caused no significant reduction in plasma viraemia (Yukl *et al.*, 2010), illustrating the possibility of on-going infection occurring in a compartment other than the blood (Table 1).

**Table 1.** Summary of the evidence for and against the hypothesis that the persistent residual viraemia in HAART-treated patients originates from a single source

Persistent viraemia originated from one source (resting T-cells)	References
• Viral genomes recovered from persistent viraemia show little variation	Bailey *et al.* (2006); Joos *et al.* (2008); Ruff *et al.* (2002)
• HAART intensification does not reduce residual viraemia	Dinoso *et al.* (2009b); Gandhi *et al.* (2010); Hatano *et al.* (2011); McMahon *et al.* (2010)
**Multiple sources contribute to persistent viraemia**	
• Persistent HIV-1 infection has been found within different parts of the body	See text
• Viral sequences distinct from those isolated from resting T-cells are found	Bailey *et al.* (2006); Brennan *et al.* (2009); Sahu *et al.* (2009)
• HAART intensification increased 2-LTR circles from PBMCs	Buzón *et al.* (2010)
• HAART intensification reduced HIV-1 RNA within the ileum	Yukl *et al.* (2010)

Most of the CD4^+^ T-cells in the body reside within the gastrointestinal tract and the lymphatic tissues rather than within peripheral blood (Mowat & Viney, 1997). In contrast, the majority of studies on HIV-1 replication dynamics and CD4^+^ T-cell depletion have been performed in peripheral blood because it is the easiest compartment to access. It has been shown that the gastrointestinal tract is the major site of HIV-1 replication and CD4^+^ T-cell depletion during all stages of HIV/AIDS (Brenchley *et al.*, 2004a; Chun *et al.*, 2008), and that the destruction of the CD4^+^ T-cell population within the gastrointestinal tract leads to the translocation of microbial products to the circulatory system and contributes to the chronic inflammation and immune exhaustion that are associated with HIV/AIDS (Douek *et al.*, 2009). Thus, we may be overlooking vital pieces of the jigsaw if we focus solely on the peripheral blood compartment.

Apart from CD4^+^ T-cells, HIV-1 can also infect cells of the monocytic lineage (Coleman & Wu, 2009; Gartner *et al.*, 1986; Le Douce *et al.*, 2010). HIV-1 infection of macrophages tends to be less cytopathic than infection of activated T-cells (Ho *et al.*, 1986, 1995; Nicholson *et al.*, 1986). Also, infected monocytes can migrate to the central nervous system and the gastrointestinal tract before maturing into macrophages, potentially sheltering the virus from the full potency of HAART (Le Douce *et al.*, 2010). The contribution of infected macrophages to HIV-related neurological decline is well documented (González-Scarano & Martín-García, 2005). It is also well known that dendritic cells can transport whole virions to lymph nodes where susceptible activated CD4^+^ T-cells reside. Moreover, dendritic cells themselves can become infected under certain circumstances (Coleman & Wu, 2009). However, it is not clear whether proviral clones or individual infected cells within the monocytic population can survive for long enough to function as long-lived latency reservoirs (Eisele & Siliciano, 2012). It is possible that within the safety of sanctuary sites and with continuous replenishment of susceptible cells, continuous productive infection may be maintained by macrophages and dendritic cells. In addition it has been proposed that infection of immature CD4^+^/CD8^+^ ‘double positive’ thymocytes during thymopoiesis may generate a population of latently infected naïve T-cells (Brooks *et al.*, 2001). Despite the continuing debate over the true origin of the low-level viraemia, it can be agreed that a viable therapeutic intervention to cure HIV/AIDS should involve the elimination of all these proven and potential reservoirs.

### Haematopoietic stem cells (HSCs) are a viral reservoir?

HSCs are a population of primitive, self-renewing precursor cells that reside in the bone marrow (Cabrita *et al.*, 2003). HSCs can proliferate and differentiate into all the cell types found in peripheral blood. HSCs and other more mature precursor cell types such as the multipotent progenitor cells (MPPs) can express the HIV-1 receptors CD4, CCR5 and CXCR4; thus in theory these cells can be infected by HIV-1 (Alexaki & Wigdahl, 2008; McNamara & Collins, 2011). However, whether this is the case *in vivo* is controversial, with contradictory evidence emerging from different studies (Davis *et al.*, 1991; Folks *et al.*, 1988; Neal *et al.*, 1995; Stanley *et al.*, 1992).

If HSCs and other progenitor cells are proven to be latent reservoirs of HIV-1, it would make the difficult task of curing HIV/AIDS even more challenging as these cells are very long-lived, can self-propagate and the provirus in these cells may not be affected by HAART or any novel therapies that target latently infected CD4^+^ T-cells. Recently, it has been shown that CD34^+^ progenitor cells (that include HSCs and MPPs) are susceptible to latent infection *ex vivo*, and that integrated provirus was detected among CD34^+^ cells from HAART-treated patients (Carter *et al.*, 2010). A follow-up study showed that only X4 or dual R5/X4-tropic viruses could efficiently infect these CD34^+^ progenitor cells (Carter *et al.*, 2011). Furthermore, this study also showed that human HSCs infected with a GFP-reporter virus could be successfully engrafted into irradiated non-obese diabetic (NOD)/SCID IL-2Rγnull mice, leading to the detection of human leukocytes in the peripheral blood that were carrying the reporter virus 14–18 weeks post-engraftment (Carter *et al.*, 2011). These results indicate that infected haematopoietic progenitor cells are a reservoir for HIV-1. However, other studies have failed to detect HIV-1 amongst the FACS-sorted CD34^+^ progenitor cells of HAART-treated patients (Durand *et al.*, 2012; Josefsson *et al.*, 2012) and it was suggested that the positive selection of CD34^+^ cells by magnetic beads as used in the studies by Carter *et al.* may be insufficient to remove all the contaminating CD4^+^ T-cells. Also, are the results from the engraftment experiment, which used a reporter virus and a highly artificial small animal model, relevant to the situation in the human body? The existence of an HSCs reservoir remains a controversy and requires further study.

## Latent HIV-1 infection of resting CD4^+^ T-cells

**Fig. 1.**
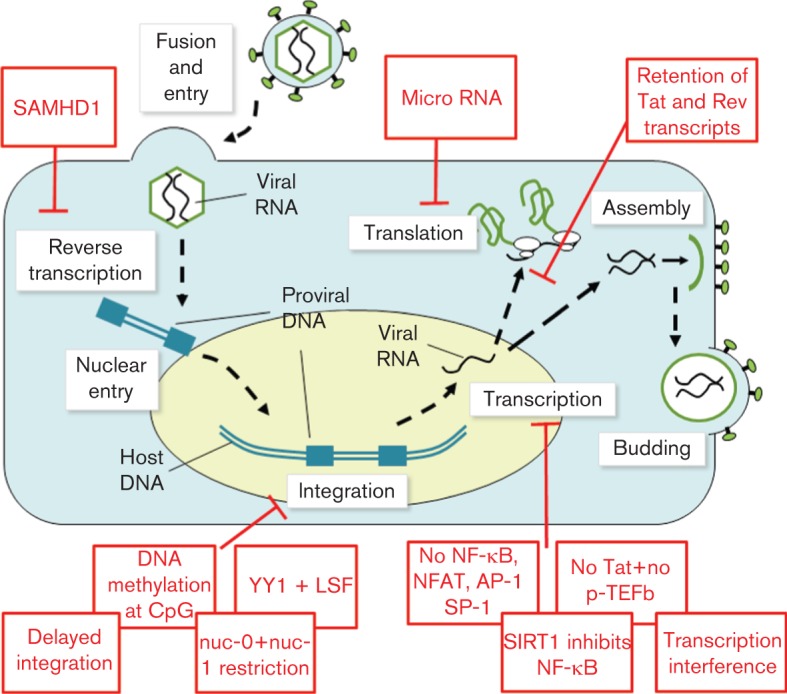
A summary of the multiple obstacles blocking productive HIV-1 infection of resting CD4^+^ T-cells. Inhibition of virus replication occurs at multiple steps during the viral life cycle. Transcription interference refers to promoter occlusion and the collision of RNA polymerases that hinder efficient viral gene expression.

Although HIV-1 can persistently replicate within sanctuary sites, improvements in drug penetration or HAART intensification may overcome this barrier to eradication in the future. However, enhancing the effectiveness of HAART will not affect the latent viruses hiding within the resting CD4^+^ T-cell populations of the body. Thus, the latent infection within resting T-cells remains the biggest proven obstacle to a sterilizing cure of HIV-1 infection. The majority of the circulating CD4^+^ T-cells in the body at any given time are in a resting state (Berard & Tough, 2002). These cells are typically defined by the lack of activation marker expression (CD25, CD69 and HLA-DR), as well as the maintenance of the cells in the G_0_ phase (Chun *et al.*, 1997). They can be broadly divided into those that have not undergone antigen-stimulated expansion (naïve T-cells) and those that have remained behind after the end of an immune response (memory T-cells) (Berard & Tough, 2002). Among infected resting T-cells, HIV-1 gene expression is largely suppressed (Hermankova *et al.*, 2003). However, some transcription of HIV mRNA can be detected within the resting T-cells of HAART-treated patients, although full virus production is inhibited by inefficiencies at various stages of the viral life cycle (Lassen *et al.*, 2004, 2006; Vatakis *et al.*, 2010) (Fig. 1). Since most of these latently infected resting CD4^+^ T-cells are CD45RO^+^ memory cells (Brenchley *et al.*, 2004b; Chomont *et al.*, 2009; Chun *et al.*, 1997; Pierson *et al.*, 2000), it is hypothesized that the majority of the latently infected T-cells come from activated CD4^+^ T-cells that were infected and then reverted back to a resting memory state before the start of virus replication (Han *et al.*, 2007). Latent provirus can be maintained within the memory T-cell population by the homeostatic proliferation of the infected host cells, driven by IL-7 (Bosque *et al.*, 2011; Chomont *et al.*, 2009).

Virus may infect resting T-cells directly and latent infection of naïve T-cells has been observed in patients, albeit at a lower frequency than memory T-cells (Chomont *et al.*, 2009; Pierson *et al.*, 2000). However, direct infection of resting T-cells is very inefficient (Pierson *et al.*, 2000; Stevenson *et al.*, 1990), with defects in reverse transcription and delays in integration in comparison with infection of activated CD4^+^ T-cells (Vatakis *et al.*, 2007, 2009). Recently, two studies have implicated the innate restriction factor SAMHD1 in the inhibition of reverse transcription in resting CD4^+^ T-cells. Initially shown to be absent in transformed CD4^+^ T-cell lines, SAMHD1 was found to be expressed in both resting and activated primary CD4^+^ T-cells. In resting T-cells, SAMHD1 restricted reverse transcription by depleting the cellular pool of dNTPs (Baldauf *et al.*, 2012; Descours *et al.*, 2012). Nevertheless, integration of the viral genome can still occur in resting T-cells (Vatakis *et al.*, 2009) and no method described to date has been able to distinguish between latently infected memory T-cells that were infected either during activation or during quiescence (Vatakis *et al.*, 2010). Furthermore, a recent study showed that in patients receiving HAART treatment, the amount of HIV DNA in memory T-cells declined over time while the amount of HIV DNA in naïve cells remained constant, suggesting that direct infection of resting T-cells may be replenishing the latent viral reservoir as the disease progresses (Wightman *et al.*, 2010). These observations are consistent with the finding that R5 tropic viruses [which are associated with acute infection (Choe *et al.*, 1996; Feng *et al.*, 1996; Zhu *et al.*, 1993)] preferentially infect CCR5-expressing memory T-cells whereas X4 tropic viruses [which are associated with late disease progression (Connor *et al.*, 1997)] exhibit a preference for CCR5- CXCR4^+^ naïve T-cells (Bleul *et al.*, 1997; Ostrowski *et al.*, 1999; Wu *et al.*, 1997). The stimulatory effects of HIV-1 gp120 may enable the direct infection of resting T-cells by activating calcium flux and NFAT signalling down the CCR5 signalling pathway, as well as upregulating inositol triphosphate-mediated signalling and the expression of the IL-2 receptor (Cicala *et al.*, 2006; Kornfeld *et al.*, 1988; Weissman *et al.*, 1997). Stimulation of CXCR4 signalling by HIV-1 gp120 induces cytoskeleton-remodelling activity in resting T-cells, increasing the efficiency of subsequent infection with HIV-1 (Yoder *et al.*, 2008). Thus, it is possible that the direct infection of resting T-cells plays an increasingly important role in maintaining the viral reservoir as the disease progresses.

The molecular mechanisms of latency in HIV-1 infection have been reviewed extensively (Coiras *et al.*, 2009; Colin & Van Lint, 2009; Marcello, 2006; Marsden & Zack, 2009; Richman *et al.*, 2009; Siliciano & Greene, 2011). In general, HIV-1 latency may be divided into pre-integration or post-integration latency. Pre-integration latency refers to the partial or complete inhibition of the viral life cycle before the integration of the virus into the host genome (see above). Most of the HIV-1 DNA found in resting T-cells is unintegrated (Chun *et al.*, 1997; Sloan & Wainberg, 2011). Although it has been shown that linear unintegrated viral DNA within resting CD4^+^ T-cells is able to complete integration after the activation of the cell (Bukrinsky *et al.*, 1991), pre-integration latency is not thought to be relevant to the establishment of the reservoir of latently infected resting T-cells due to the labile nature of viral DNA in the cytoplasm of the cell (Pierson *et al.*, 2002). Accordingly, the unintegrated viral DNA may no longer be replication competent after a protracted period inside the host cell (Han *et al.*, 2007; Zhou *et al.*, 2005). Post-integration latency is the failure of expression of the viral genome after it has been integrated into the host genome. While less than 0.05 %, or approximately 10^6^ to 10^7^ cells, carry integrated provirus, it is these integrated proviruses that are thought to constitute the latent viral reservoir (Chun *et al.*, 1997).

Even after a successful integration event, there are still multiple barriers to productive HIV-1 replication within resting CD4^+^ T-cells (Fig. 1). Although HIV-1 genomes are generally integrated into genes that are actively expressed within resting T-cells (Han *et al.*, 2004), viral gene expression may be downregulated by promoter occlusion (if the provirus is integrated in the same orientation as the host gene) (Greger *et al.*, 1998) or by collisions between RNA Pol II molecules that are travelling in opposite directions (if the provirus is integrated in the opposite orientation as the host gene) (Han *et al.*, 2008). Two nucleosomes, named nuc-0 and nuc-1, are frequently associated with the HIV-1 5′LTR and regulate the basal transcriptional activity of the viral genome by controlling the access of transcription factors to the LTR (Verdin *et al.*, 1993). The remodelling of the nucleosomes is regulated by the acetylation status of their constituent histones, which is in turn controlled by enzymes such as histone acetyltransferases and histone deacetylases (Van Lint *et al.*, 1996). This allows the manipulation of HIV-1 transcriptional activity by pharmacological means, potentially leading to a viable method to eliminate the virus reservoir from resting CD4^+^ T-cells (see Novel drug discovery). The presence of cellular transcriptional repressors, for example YY1 and LSF, as well as the methylation of the two CpG islands at the HIV-1 transcription start site, can recruit histone deacetylases to the HIV-1 LTR and reinforces latency (Blazkova *et al.*, 2009; Coull *et al.*, 2000; Kauder *et al.*, 2009), while the binding of the transcription factor NF-κB stimulates proviral reactivation by recruiting histone acetyltransferases to the LTR and initiating early HIV-Tat production (Lusic *et al.*, 2003; Williams *et al.*, 2007). The lack of NF-κB, as well as transcription factors NFAT, SP-1 and AP-1 prevents the synthesis of Tat and the subsequent Tat-dependent, high level viral gene expression (Coiras *et al.*, 2009; Mbonye & Karn, 2011; Williams & Greene, 2007). The transcriptional activity of Tat is highly dependent on interacting with the cellular factor P-TEFb, which triggers effective RNA Pol II elongation (Parada & Roeder, 1996), and the negative regulation of P-TEFb activity in resting T-cells further impairs the expression of HIV-1 genes (Ghose *et al.*, 2001). Tat also interacts with several other cellular factors such as the histone acetyltransferases p300 and P/CAF to promote transactivation of viral genes (Benkirane *et al.*, 1998). The acetylation of the RelA subunit of NF-κB by p300 increases its transcriptional activity (Chen *et al.*, 2002) and this is countered by the cellular deacetylase SIRT1 (Yeung *et al.*, 2004). SIRT1 activity is in turn blocked by HIV-1 Tat (Kwon *et al.*, 2008). In addition, it has been demonstrated that HIV Tat and Rev transcripts are retained in the nuclei of resting CD4^+^ T-cells (Lassen *et al.*, 2006) and that numerous host microRNAs can directly or indirectly downregulate HIV-1 gene expression, contributing to the maintenance of proviral latency (Chiang & Rice, 2012).

Due to the involvement of so many cellular factors, it has been proposed that there are different degrees of latency within the T-cell population, depending on the cell type and the extracellular environment (Pace *et al.*, 2011). A recent *in vitro* study of HIV-1 latency using a central memory T-cell model system has shown that IL-7-driven homeostatic replication of infected cells can induce partial virus reactivation, while stimulation of the T-cell receptor signalling pathway with anti-CD3/anti-CD28 antibody induced full reactivation (Bosque *et al.*, 2011). This supports the hypothesis of a dynamic reservoir of infected T-cells at various levels of cellular and viral activation.

An area of research which has, as yet, escaped the attention of the HIV-latency field is the molecular mechanism behind CD4^+^ T-cell quiescence. It has been known for some time that the quiescence state is actively maintained by factors such as LKLF, Tob, Foxo3a and Foxj1 (Coffer & Burgering, 2004; Tzachanis *et al.*, 2004; Yusuf & Fruman, 2003). The role of these factors in HIV-latency has been explored by few laboratories so far (Haaland *et al.*, 2005; van Grevenynghe *et al.*, 2008) and further research may provide new insights into the mechanism of latency as well as potential therapeutic targets.

## *In vitro* and *in vivo* models of latency

The latently infected CD4^+^ T-cell population within the patient is very small, thus making *ex vivo* experiments very difficult. The use of *in vitro* and *in vivo* models of latency has been and will continue to be vital to the understanding of HIV-1 latency and drug discovery. Early studies of lentiviral latency using cell lines such as ACH-2, U1 and J-Lat showed the involvement of host cytokine signalling pathways and chromatin reorganization in modulating latency (Folks *et al.*, 1987, 1989; Jordan *et al.*, 2003), but their transformed nature means their responses to treatments may not be physiologically relevant. For example, in the latently infected J-Lat cell line, HIV-1 preferentially integrates near the heterochromatin where transcriptional activity is low (Jordan *et al.*, 2003). However, this preference is not observed within the latently infected resting CD4^+^ T-cells from HAART-treated patients, rather the provirus overwhelmingly favours integration into active transcriptional regions (Han *et al.*, 2004; Schröder *et al.*, 2002).

Most of the current *in vitro* models of HIV-1 latency involve the use of primary cells (Yang, 2011). These experiments are technically challenging, often taking weeks or months to complete in order to mimic the transition of activated T-cells to quiescent memory T-cells *in vivo* (Marini *et al.*, 2008). Generating enough cells for experiments, especially in high-throughput screening of compounds, is another problem. Strategies such as the transduction of a survival gene into primary cells (Yang *et al.*, 2009), using low levels of cytokines such as IL-2 or IL-7 (Bosque & Planelles, 2009; Marini *et al.*, 2008) or co-culture with a feeder cell line (Sahu *et al.*, 2006; Tyagi *et al.*, 2010) have been described. Protocols to directly infect purified resting T-cells *ex vivo* have also been developed. To overcome the inefficient nature of infecting resting T-cells, methods such as spinoculation (O’Doherty *et al.*, 2000) or stimulation with the chemokines CCL19 and CCL21 (Saleh *et al.*, 2007, 2011) were used. The pros and cons of these *in vitro* model systems have been reviewed elsewhere (Pace *et al.*, 2011; Wightman *et al.*, 2012; Yang, 2011). Any model of latency would have to balance multiple conflicting demands such as maintaining the viability of the cells, while preserving a resting state and allowing viral integration without stimulating full-blown virus replication. A further complication is the fact that there are multiple types of cells that can be latently infected, such as central memory T-cells, transitional memory T-cells and naïve T-cells (Chomont *et al.*, 2009; Wightman *et al.*, 2010); any future treatments to reactivate the latent proviruses would have to be effective in all of these subsets of latently infected T-cells.

Non-human primates, in particular rhesus macaques infected with the simian immunodeficiency virus (SIV) or chimeric SIVs containing HIV-1 reverse transcriptase have been used to model HIV-1 latency in HAART-treated patients (Dinoso *et al.*, 2009a; North *et al.*, 2010; Shen *et al.*, 2003). The major advantage of using non-human primates is that the locations of the persistent viral reservoirs mirror those in humans (North *et al.*, 2010), which allows comparative *in vivo* studies. Also the progression of SIV in macaques resembles HIV-1 infection in humans, with distinctive acute and chronic phases of infection that may lead to immunodeficiency (Hirsch *et al.*, 1996). However, there are significant differences between SIV infection of non-human primates and HIV-1 infection in humans. For example, the residual viraemia for SIV in rhesus macaques during chronic infection is higher than the levels seen in humans (Brenchley & Paiardini, 2011). The progression to AIDS appears to be more rapid in rhesus macaques than in humans (North *et al.*, 2010). In African green monkeys or sooty mangabeys, although high levels of virus replication are observed during the chronic phase of infection, this is not accompanied by the destructive chronic immune activation seen in rhesus macaques or humans (Brenchley & Paiardini, 2011; Chahroudi *et al.*, 2012). Also different strains of SIV can produce different pathologies in the same host (Hirsch *et al.*, 2000).

The complexity of finding the correct host and SIV strain combination that mimics HIV-1 latent infection most closely, together with issues such as ethical concerns and high cost have led to the development of other, non-primate animal models for HIV-1 infection such as humanized SCID (SCID-hu) mouse models (Boberg *et al.*, 2008; Brooks *et al.*, 2001; Van Duyne *et al.*, 2009). SCID-hu mice are created by transplanting SCID mice with human fetal thymus and liver tissues or peripheral blood lymphocytes to form SCID-hu Thy/Liv and SCID-hu PBL mice, respectively (Van Duyne *et al.*, 2009). For example an *in vitro* model of latently infected immature CD4^+^/CD8^+^ thymocytes has been generated using SCID-hu Thy/Liv mice (Brooks *et al.*, 2001; Burke *et al.*, 2007). A major drawback of using SCID-mouse-based models is the failure to fully reconstitute the human immune system within the transplanted animals (Rossi *et al.*, 2001; Van Duyne *et al.*, 2009). Further improvement to efficiency of engraftment was achieved with the generation of the NOD/SCID mouse model (Hesselton *et al.*, 1995) and later with the double knockout of the common cytokine receptor γC and the recombinase activating gene 2 (Rag2) (Goldman *et al.*, 1998). The transplantation of human CD34^+^ stem cells into Rag2^−/−^γC^−/−^ mice leads to the development of a functional model of the human immune system in the bodies of the mice (Traggiai *et al.*, 2004) and forms the basis of a recent murine model of HIV-1 latency that contains infected resting T-cells in the peripheral blood and lymphoid tissues (Choudhary *et al.*, 2009; 2012). Viable small animal models are vital in the preclinical evaluation of latency reversing therapies, especially if they can replicate latent infection compartments other than peripheral blood.

### The feline model of HIV-1 latency

Feline immunodeficiency virus (FIV) was discovered in 1986 in California (Pedersen *et al.*, 1987). Both HIV-1 and FIV target activated CD4^+^ T-cells (Yamamoto *et al.*, 1988; Zagury *et al.*, 1986), but whereas the primary receptor for HIV-1 is CD4 (Dalgleish *et al.*, 1984), the primary receptor for FIV is CD134 (OX40) (Shimojima *et al.*, 2004). HIV-1 utilizes CCR5 and CXCR4 as its secondary receptors (Choe *et al.*, 1996; Deng *et al.*, 1996; Feng *et al.*, 1996), while FIV uses CXCR4 alone as its sole secondary receptor (Willett *et al.*, 1997). FIV is transmitted mainly by biting (Yamamoto *et al.*, 1989) and causes clinical signs in cats that are similar to AIDS in humans (Ackley *et al.*, 1990; Barlough *et al.*, 1991; Novotney *et al.*, 1990).

Since FIV has a similar cell tropism to HIV-1, it is expected that the host response to FIV and its pathogenesis will be comparable to HIV-1. Cats mount both humoral and cytotoxic T-cell responses to FIV infection (Beatty *et al.*, 1996; Egberink *et al.*, 1992; Flynn *et al.*, 2002). However, the hosts usually fail to clear the infection and may succumb to immunodeficiency. The mechanisms of pathogenesis of HIV-1 and FIV are remarkably similar. Both viruses cause massive depletion of the gastrointestinal tract CD4^+^ T-cell population (Brenchley *et al.*, 2004a; Howard *et al.*, 2010). The low fidelity of the HIV-1 and FIV reverse transcriptases results in the generation of a diverse pool of viral variants within the host, encouraging immune escape (Bebenek *et al.*, 1993; Hosie *et al.*, 2011; Kraase *et al.*, 2010; Mansky & Temin, 1995). All of these factors promote chronic immune activation, eventually leading to the breakdown of the host immune system (Douek *et al.*, 2009; Tompkins & Tompkins, 2008).

Previous studies of FIV in cats have shown that activated (CD4^+^ CD25^+^) and resting (CD4^+^ CD25^–^) CD4^+^ T-cells from peripheral blood can be latently infected *ex vivo* and that FIV replication can be reactivated by the application of ConA or IL-2 (Joshi *et al.*, 2005, 2004), mirroring the crucial role of IL-2 in productive infection with HIV-1 (Oswald-Richter *et al.*, 2004). In a separate study, cats challenged with a low-dose exposure to FIV-infected T-cells showed an aviraemic infection, and when cells from multiple tissues were stimulated by PMA, FIV gp120 production was detected (Assogba *et al.*, 2007). More recently it has been shown that FIV establishes a latent infection within activated and resting T-cells of cats during the asymptomatic phase of infection, similar to the latent infection of the resting T-cell population by HIV-1 in humans (Murphy *et al.*, 2012). These cells contained detectable FIV DNA but no FIV RNA. Furthermore, virus replication from these latently infected cells could be reactivated *ex vivo* by the mitogens PHA and PMA as well as the histone deacetylase inhibitor SAHA (McDonnel *et al.*, 2012) (see Novel drug discovery). These findings support the proposal of using FIV-infected cats as an alternative small animal model for HIV-1 latency.

## Stem cell transplantation and gene therapy approaches to curing HIV/AIDS

Recently, a HIV-1-positive patient who developed acute myeloid leukaemia was apparently cured of HIV-1 infection after receiving an HSC transplant from a donor who was homozygous for the CCR5 Δ32 allele (Hütter *et al.*, 2009). The patient underwent intensive chemotherapy and radiotherapy to prepare for the transplant, which presumably also eliminated almost all the infected CD4^+^ T-cells within the body. In addition, the patient developed graft-versus-host disease after transplantation, indicating that the transplanted cells had replaced the host immune system. HAART treatment was then stopped and in the follow-up study the patient was shown to remain free from the virus (Allers *et al.*, 2011). To subject otherwise healthy HAART-treated patients to this potentially lethal procedure is ethically questionable and practically not viable, especially in resource-poor settings. However, this unique case has raised an interesting question regarding the kind of intervention necessary to clear the body of HIV-1: was the cure achieved by the intensive chemotherapy and radiotherapy or by the transplant of the Δ32 HSCs, which gave rise to HIV-1 resistant CD4^+^ T-cells? Treatment of HIV-1-positive lymphoma patients with autologous stem cell transplants failed to eliminate the virus from the body (Cillo *et al.*, 2012), which indicated the presence of residual virus or infected cells within the extracted autologous cell population or within the host. It also demonstrated the need to make the host CD4^+^ T-cells immune to HIV-1 infection before transplantation.

The CCR5 Δ32 mutation abrogates infection of CD4^+^ T-cells by R5 HIV-1 viruses (Dean *et al.*, 1996), the strains most frequently associated with early stage infection and which are transmitted preferentially between individuals (Margolis & Shattock, 2006). Thus, the nascent CD4^+^ T-cells from the transplant would be resistant to new infection. Disruption of the CCR5 gene has no apparent undesirable effects on the normal functioning of HSCs (Bai *et al.*, 2000; O’Brien & Moore, 2000). Various techniques have been developed to disrupt the CCR5 gene *ex vivo*, including the use of CCR5-specific siRNAs, ribozymes, intrabodies and zinc-finger nucleases (ZFNs) (Anderson *et al.*, 2007; Bai *et al.*, 2000; Holt *et al.*, 2010; Kumar *et al.*, 2008; Swan *et al.*, 2006). Each of these treatments has been tested in mouse models and led to the production of modified HSCs which give rise to CD4^+^ T-cells that are resistant to R5 HIV-1 infection. ZFNs, which are engineered endonucleases containing zinc finger domains that recognize specific DNA sequences (Urnov *et al.*, 2005), have also been used to disrupt the CCR5 gene in CD4^+^ T-cells in a mouse model of HIV-1 infection (Perez *et al.*, 2008). More recently, ZFNs targeting CD4^+^ T-cells have been successfully tested in a phase I clinical trial, in which the treatment was well tolerated by patients, the modified CD4^+^ T-cells were able to persist in the body and there were improvements on the CD4^+^ T-cell count and CD4^+^:CD8^+^ T-cell ratio (June *et al.*, 2012). ZFNs that can disrupt the CXCR4 gene in CD4^+^ T-cells have also been developed and it has been demonstrated that they confer resistance to cells against the X4-tropic HIV-1 strains associated with late-stage infection (Wilen *et al.*, 2011; Yuan *et al.*, 2012). Combining the disruption of CCR5 and CXCR4 may provide a viable gene therapy approach to a functional cure, in which the patient’s CD4^+^ T-cells are made resistant to HIV-1 *ex vivo* and are reintroduced back into the body. There may still be residual viraemia but the virus would not cause disease after the withdrawal of HAART. Potential problems with the use of the ZFNs include the possibility of non-specific cleavage of host DNA (Gabriel *et al.*, 2011; Pattanayak *et al.*, 2011) and the possibility of adverse effects from disrupting CXCR4, which has not been well studied at the time of writing.

## Novel drug discovery

The main strategy that is currently being pursued by many laboratories to eradicate HIV-1 from the body is to reactivate the latent virus reservoir within resting CD4^+^ T-cells (Marsden & Zack, 2009; Richman *et al.*, 2009). Early attempts at reactivation using powerful cytokines such as IL-2 and TNF-α stimulated virus production (Chun *et al.*, 1998) but also caused dangerous side effects such as the non-specific, global activation of T-cells (Prins *et al.*, 1999). In contrast, the cytokine IL-7 has also been shown to have potent anti-HIV latency effects without inducing T-cell activation (Levy *et al.*, 2009; Scripture-Adams *et al.*, 2002; Wang *et al.*, 2005). IL-7 is well-tolerated *in vivo* (Levy *et al.*, 2012) and it has been used in a clinical trial to reduce the latent reservoir size (ERAMUNE 01, due to finish in January 2013. http://clinicaltrials.gov). However, a potential problem with the use of IL-7 to deplete the latent reservoir is that at low concentrations, IL-7 can promote the survival or induce the homeostatic proliferation of the latently infected memory T-cells without triggering activation of the virus, thus inadvertently expanding the reservoir of infected cells (Chomont *et al.*, 2009; Marini *et al.*, 2008).

Compounds that stimulate protein kinase C and NF-κB such as the phorbol ester prostratin and 5-hydroxynaphthalene-1,4-dione (5HN) have been shown to reactivate latent infection *in vitro* (Kulkosky *et al.*, 2001; Yang *et al.*, 2009). Intriguingly, prostratin also has anti-HIV-1 replication effects (Biancotto *et al.*, 2004; Rullas *et al.*, 2004) and a similar dual effect of the compound on FIV replication has been described *in vitro* (Chan *et al.*, 2013). Histone deacetylase inhibitors such as valproic acid and suberoylanilide hydroxamic acid (SAHA or Vorinostat) have been shown to reverse HIV latency by remodelling the HIV-repressive nucleosome nuc-1 (Archin *et al.*, 2009; Contreras *et al.*, 2009; Van Lint *et al.*, 1996; Ylisastigui *et al.*, 2004). SAHA is a selective class I and II histone deacetylase inhibitor and is approved as a clinical treatment for cutaneous T-cell lymphoma. It has been used in a number of *ex vivo* and clinical studies of the latent reservoir (Archin *et al.*, 2012; Shan *et al.*, 2012). However, further research is required to investigate fully the long-term side effects of SAHA in terms of its potential as a mutagen and its ability to reactivate other latent viruses (Archin *et al.*, 2012; Kerr *et al.*, 2010; Wightman *et al.*, 2012). Using a siRNA screen, a novel HIV replication-inhibiting host factor has been identified recently (Zhu *et al.*, 2012). This factor, named bromodomain containing 4, can be inhibited by a small molecule known as JQ1 (Filippakopoulos *et al.*, 2010) and JQ1 has been shown to have HIV-1 latency reversing activity by several laboratories (Banerjee *et al.*, 2012; Li *et al.*, 2013; Zhu *et al.*, 2012). Other compounds and small molecules that have been shown recently to reactivate latent HIV-1 infection include a bacterial protein named HIV-1-reactivating factor (Wolschendorf *et al.*, 2010), the aldehyde dehydrogenase inhibitor disulfiram (Xing *et al.*, 2011) and a number of quinolin-8-ol derivatives (Xing *et al.*, 2012). Curiously, a recent clinical trial has demonstrated that intensification of HAART with the CCR5 antagonist Maraviroc (MVC) caused a reduction in the size of the latently infected T-cell reservoir (Gutiérrez *et al.*, 2011). The mechanism behind this effect of MVC is unknown, but the binding of MVC to CCR5 may lead to the stimulation of cell signalling analogous to the stimulation of the other major HIV-1 co-receptor, CXCR4, by the binding of HIV-1 Env (Wu & Yoder, 2009; Yoder *et al.*, 2008). If the findings of this study are confirmed, a new way of reactivating latently infected cells may have been identified. Moreover, it has been shown recently that fever enhances the activity of Tat, a phenomenon mediated by the heat-shock protein Hsp-90 (Roesch *et al.*, 2012). Using a J-Lat model, this study demonstrated that although hyperthermia by itself cannot reactivate latency, it can enhance the reactivation effect of other treatments such as the co-cultivation of the J-Lat cells with IL-2 supplemented PBMCs (Roesch *et al.*, 2012). This suggests that the artificial induction of fever may be used to boost the effectiveness of any future latency reversing therapies.

Borrowing from the concept of HAART, a combination of different latency reversing agents may be used synergistically to enhance the effect of reactivation therapies (Burnett *et al.*, 2010; Deeks *et al.*, 2012; Reuse *et al.*, 2009). The number of compounds identified is likely to increase thanks to on-going and future high-throughput screens that look for molecules which can stimulate latent HIV-1 to reactivate. One novel screening method described recently can measure the expression of cell-associated viral RNA among latently infected T-cells as soon as the cells are extracted from the patient without the need for further co-culturing (Archin *et al.*, 2012). Using this assay, an increase in viral RNA can be demonstrated amongst the resting T-cells from patients after being treated with a single dose of SAHA. However, an important caveat to this type of experiment is that stimulation of viral transcription, viral protein synthesis or even virion production may not necessarily lead to the destruction of the latently infected cell (see below).

## Stimulation of latent virus replication may not lead to the depletion of the viral reservoir

**Fig. 2.**
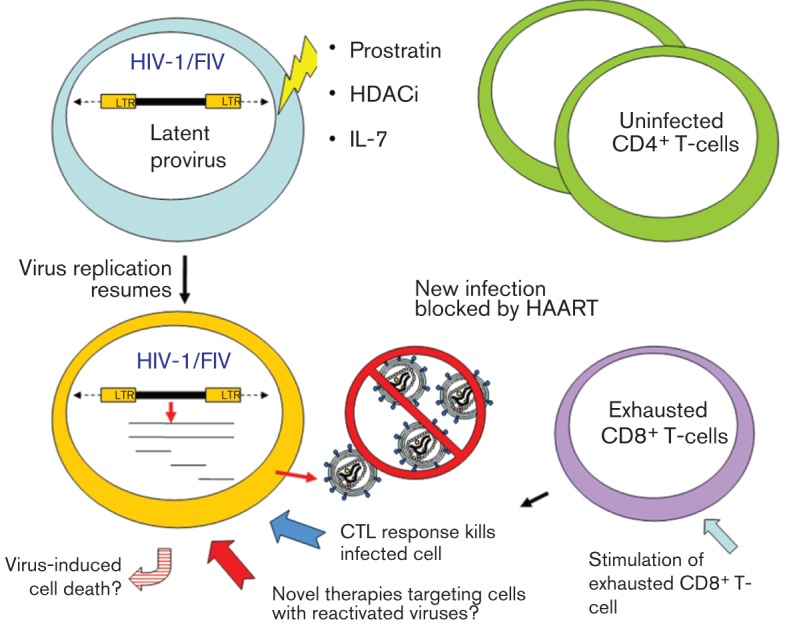
A theoretical scheme to eliminate the latent HIV-1 reservoir within the resting T-cell population. Virus replication in latently infected cells could be reactivated by, for example, treatments with prostratin, histone deacetylase inhibitors (HDACi) or IL-7. Meanwhile the CTL responses of the patient could be restored by stimulation with viral antigens and cytokines. Although the reactivation of the latent virus may not lead to the apoptosis of the infected cell as previously assumed, the restored CTL response or the use of novel drug-delivery technologies may allow the specific targeting of the infected cells for destruction.

It has been assumed that once the latent provirus is reactivated inside a resting T-cell, the cell would die by HIV-induced cytopathic effects or be killed by the host immune response (Richman *et al.*, 2009). However, this view has recently been challenged (Shan *et al.*, 2012). Stimulation of resting CD4^+^ T-cells from HAART-treated patients with the SAHA did not reduce the size of the latent reservoir (Shan *et al.*, 2012). Furthermore, latently infected resting T-cells reactivated by SAHA were killed neither by viral cytopathic effects, nor by autologous CD8^+^ T-cells isolated from the same patients. Only after antigen-specific stimulation of the autologous CD8^+^ T-cells was efficient killing of the SAHA-reactivated, infected resting CD4^+^ T-cells restored. The transduction of the survival gene Bcl-2 into the resting T-cells during the establishment of the latent infection assay, as well as the use of modified reporter viruses may have increased the survival rate during the study. However, reactivation by SAHA of the latent wild-type virus within unmodified resting T-cells isolated from patients also did not lead to a contraction of the latent virus reservoir. These findings showed that any future therapeutic regime to eliminate the latent reservoir would require the boosting of anti-HIV cytotoxic T-lymphocyte (CTL) responses, which would likely be in a state of exhaustion after years of chronic activation (Trautmann *et al.*, 2006). In addition to stimulating the CD8^+^ T-cells with viral antigens and cytokines, inhibiting the function of immunoregulatory molecule such as PD-1 may be another option for the restoration of full CTL function in the patient against HIV-1 (Eichbaum, 2011). Also, it is known that resting T-cells are less vulnerable to cell death compared with their activated counterparts (van Leeuwen *et al.*, 2009). Would the use of drugs that stimulate cellular activation, such as prostratin, lead to the death of the reactivated infected T-cells? Alternatively, is it possible to use novel technologies such as nanoparticles (Peer *et al.*, 2007), intrabodies (Pérez-Martínez *et al.*, 2010) or RNA aptamers (Burnett & Rossi, 2012) to target the reactivated infected T-cells for destruction (Fig. 2)?

Another observation that may be a cause for concern among the ever growing literature on latency-reversing compounds is that even the most promising molecules such as prostratin, SAHA and JQ1 cannot reliably stimulate productive infection from all HAART-treated patients’ samples, despite being very successful in reactivating latent viruses from *in vitro* models (Contreras *et al.*, 2009; Kulkosky *et al.*, 2001; Zhu *et al.*, 2012). The variable performances of these compounds may be due to sampling errors as a result of the fact that there are so few latently infected cells within the patient (hence the need for *in vitro* model systems), or the underlying activation status of the cells, as in the case for prostratin (Chan *et al.*, 2012; Kulkosky *et al.*, 2001). Alternatively, this may indicate that the current *in vitro* models does not represent all the subset of CD4^+^ T-cells that are latently infected. Also can we assume that our current methods of handling CD4^+^ T-cells accurately reproduce *in vivo* conditions? Nevertheless, the potential for false negatives and false positives in the current assays demands further research into the basic molecular biology of HIV-1, T-cell biology and improvements to existing HIV-latency models.

### Conclusion

After more than two decades of research we are only beginning to appreciate the full complexity of the problem of HIV-1 persistence and latency. Recent research suggests that there are multiple reservoirs of replication-competent virus which contribute to viral persistence. To achieve a sterilizing cure of HIV-1 requires significant disruption or even elimination of all these reservoirs. In addition, there are still many unanswered questions regarding HIV-1 latency remaining. For example, what is the source of the persistent low-level viraemia? What is the contribution of direct infection of resting T-cells to the overall size of the viral reservoir? Can gene therapy lead to a functional cure of HIV-1? How do we eliminate the infected T-cells once they are reactivated? Research into novel small animal models of HIV-1 latency such as the Rag2^−/−^γC^−/−^ mouse or FIV-infection of cats may speed up the drug development process but their relevance to the clinic needs to be established. Further research into these issues is needed urgently in order to stop the global HIV/AIDS epidemic, which continues to be a serious global threat to public health almost 30 years after its discovery.
